# Automatic Actin Filament Quantification of Osteoblasts and Their Morphometric Analysis on Microtextured Silicon-Titanium Arrays

**DOI:** 10.3390/ma5071176

**Published:** 2012-06-27

**Authors:** Claudia Matschegewski, Susanne Staehlke, Harald Birkholz, Regina Lange, Ulrich Beck, Konrad Engel, J. Barbara Nebe

**Affiliations:** 1 Biomedical Research Center, Department of Cell Biology, University of Rostock, Schillingallee 69, 18057 Rostock, Germany; E-Mails: claudia.matschegewski@uni-rostock.de (C.M.); susanne.staehlke@uni-rostock.de (S.S.); 2 Institute of Mathematics, University of Rostock, Ulmenstrasse 69, 18057 Rostock, Germany; E-Mails: harald.birkholz@uni-rostock.de (H.B.); konrad.engel@uni-rostock.de (K.E.); 3 Department of Electrical Engineering and Informatics, University of Rostock, A.-Einstein-Strasse 2, 18059 Rostock, Germany; E-Mails: regina.lange@uni-rostock.de (R.L.); ulrich.beck@uni-rostock.de (U.B.)

**Keywords:** actin cytoskeleton, MG-63 osteoblasts, microtexturing, ridge detection, image processing

## Abstract

Microtexturing of implant surfaces is of major relevance in the endeavor to improve biorelevant implant designs. In order to elucidate the role of biomaterial’s topography on cell physiology, obtaining quantitative correlations between cellular behavior and distinct microarchitectural properties is in great demand. Until now, the microscopically observed reorganization of the cytoskeleton on structured biomaterials has been difficult to convert into data. We used geometrically microtextured silicon-titanium arrays as a model system. Samples were prepared by deep reactive-ion etching of silicon wafers, resulting in rectangular grooves (width and height: 2 µm) and cubic pillars (pillar dimensions: 2 × 2 × 5 and 5 × 5 × 5 µm); finally sputter-coated with 100 nm titanium. We focused on the morphometric analysis of MG-63 osteoblasts, including a quantification of the actin cytoskeleton. By means of our novel software FilaQuant, especially developed for automatic actin filament recognition, we were first able to quantify the alterations of the actin network dependent on the microtexture of a material surface. The cells’ actin fibers were significantly reduced in length on the pillared surfaces *versus* the grooved array (4–5 fold) and completely reorganized on the micropillars, but without altering the orientation of cells. Our morpho-functional approach opens new possibilities for the data correlation of cell-material interactions.

## 1. Introduction

In implant technology, topographical surface modification has been demonstrated to be a powerful tool to optimize implant designs for clinical applications [1,2]. Because of its great potential, numerous biological studies focus on the investigation of topographical effects on cellular physiology [3,4,5]. However, the mechanisms involved in the differential cell physiology in response to surface microtexture are not well understood and current data concerning quantitative relationships between these parameters is still unsatisfactory. Therefore a detailed clarification of the fundamental principles in the cell-material dialogue is in great demand.

When cells attach to a biomaterial, their functional behavior depends on its physico-chemical properties, which are determining factors for the quality of the cell-material interaction [6]. Thereby surface microtexture has been shown to possess a remarkable influence on diverse cellular functions, among them adhesion and morphology, integrin expression and spreading; these are known to further affect the cell physiology and proliferation [7,8]. Thus, these parameters represent useful investigation tools with regard to their distinct interactions with the surface [4]. Moreover it has been shown that actin organization can also be affected by surface structure; here, studies demonstrate a surface topography-dependent alteration of actin filament formation in osteoblasts grown on substrata with defined surface topography [9,10]. Actin is the major cytoskeletal component and plays a central role in important cellular processes, e.g., in transduction of information in the outside-in signaling of cells by transmitting extracellular forces and tensions, and thus influencing the cell morphology [11,12]. In particular, many studies report that the arrangement of the actin cytoskeleton is decisive for cellular spreading as well as for the length control of cells [13,14].

In view of the high complexity of the cell-material interactions, it is extremely difficult to specify the impact of one factor without influencing the others. In many biological analyses of the behavior of osteoblasts dependent on the biomaterial’s surface topography, titanium substrates are used due to their biocompatibility and major relevance for applications in hard tissue implants [15]. Research in this regard is mainly performed on randomly micro-roughened titanium surfaces [16,17], and is based on observations of a better bone-to-implant interaction including an accelerated osseointegration on rougher surfaces as compared to smooth ones [6,15]. Beyond these stochastic roughness modifications, controlled microtexturing can induce topography-dependent cell alignment (contact guidance) and directed cell migration [18,19]. Investigations on defined microstructured dental implants showed that they prevent epithelial downgrowth or increase pull-out strength [5]. Thus, controlled surface patterning might be a promising tool for directing cell behavior and guiding tissue-implant integration. Moreover, in order to discriminate between specific topographical effects, the fabrication of surfaces with controlled topographical textures might facilitate cell-material data correlation due to the fact that the data can be well described by only a limited set of characteristic parameters in comparison to stochastic surfaces.

When considering the entire scientific work on cellular behavior on structured material surfaces, it becomes apparent that finding correlations between surface characteristics and the behavior of the biosystem is one of the most important challenges in implant technology.

In order to enhance the current state of data concerning relationships in cell-material interactions, we tried to determine and further quantify additional cellular parameters by means of mathematical modeling based on confocal microscopic images, and thus extend the spectrum of cell biological parameters. Mathematical image processing facilitates the quantitative assessment of specific aspects of cellular behavior with minimal manual input. Its automation allows for the recognition of visual features in a trade-off between tolerance for imaging modalities and accuracy of meaningful parameters. The quality of connectivity information seems to be improved, compared to state of the art methods for different kinds of images [20,21].

In our study we focused on the quantification of the cellular phenotype and the actin filament formation of osteoblasts growing on geometrically microtextured silicon-titanium arrays to determine their influence on cell-morphometric parameters. For this purpose microscopic images from scanning electron microscopy and confocal microscopy were analyzed. MG-63 osteoblasts were used as model cells because of their similar cell physiological behavior to primary bone cells, like already revealed in our previous studies with stochastically modified surfaces [3,17]. By means of mathematical modeling for the quantification of the cellular actin cytoskeleton we wanted to find relationships between the organization of intracellular structures and the microtexture of the surface. In particular, our mathematical approach for image processing provides automated analysis of the cell parameter actin filament formation, including filament length, filament number and orientation dispersion, thus broadening the scope of acquisition of biological data for the data correlation of cell *vs.* material characteristics.

## 2. Materials and Methods

### 2.1. Titanium Arrays

**Figure 1:**
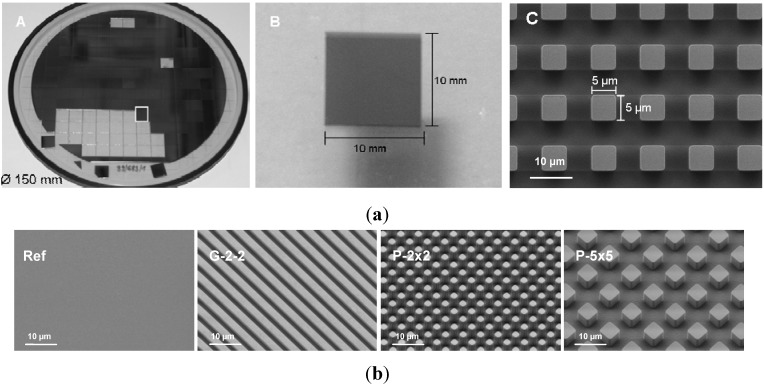
(**a**) Size and dimension of fabricated samples. (A) wafer Ø150 mm with arrays 10x10 mm, (B) single array 10 × 10 mm and (C) FE-SEM image of Ti-coated periodical cubic pillar array with the dimension 5 × 5 × 5 µm (P-5×5) (FE-SEM Supra 25, Carl Zeiss, bar = 10 µm). (**b**) FE-SEM images of Ti-coated periodical arrays on silicon substrate with regular geometry: planar titanium reference (Ref), rectangular grooved array of 2 µm width and 2 µm height (G-2-2), cubic pillar arrays with the dimensions 2 × 2 × 5 µm (P-2×2) and 5 × 5 × 5 µm (P-5×5) (FE-SEM Supra 25, Carl Zeiss, bar = 10 µm).

For the experiments, periodically microtextured samples with different regular surface geometry were used. For sample fabrication, silicon wafers with a diameter of 150 mm and a thickness of 500 µm were microstructured using deep reactive-ion etching (DRIE) (Center for Microtechnologies ZFM, Chemnitz, Germany) (Figure 1a) [18,22]. The fabricated samples (sizing 10 × 10 mm) possess three distinct regular surface geometries: (i) periodically grooved topography with a plateau and groove width of 2 µm and a step height of 2 µm (G-2-2), (ii) regular cubic pillar geometry in two different dimensions with pillars of 2 × 2 × 5 µm (P-2×2) and 5 × 5 × 5 µm (P-5×5) and a pitch width of 4 µm and 10 µm, respectively and (iii) unstructured planar silicon wafers as control (Ref). Finally, the samples were sputter-coated with 100 nm titanium. Qualitative analysis of the samples was made using field-emission scanning electron microscopy (FE-SEM Supra 25; Carl Zeiss, Jena, Germany) (Figure 1b).

### 2.2. Cell Culture

Titanium arrays were washed in 70% ethanol for 15 min, rinsed in phosphate-buffered saline (PBS) (PAA Laboratories, Pasching, Austria) and then placed into 4-well NUNC dishes (Thermo Fisher Scientific, NUNC GmbH & Co. KG, Langenselbold, Germany). Human osteoblastic cells (MG-63, ATCC, CRL-1427) were seeded at a density of 3 × 10^4^ cells/array in Dulbecco’s modified Eagle medium (DMEM) (Invitrogen GmbH, Karlsruhe, Germany), containing 10% fetal calf serum (FCS) (PAA Laboratories, Pasching, Austria) and 1% gentamicin (Ratiopharm GmbH, Ulm, Germany) at 37 °C and in a humidified atmosphere with 5% CO_2_.

### 2.3. Cell Morphology Visualized by FE-SEM

MG-63 cells were grown on the titanium arrays for 24 h, fixed with 2.5% glutaraldehyde (1 h, 4 °C), dehydrated through a graded series of acetone (30% 5 min, 50% 5 min, 75% 10 min, 90% 15 min, 100% twice for 10 min) and dried in a critical point dryer (K 850, EMITECH, Taunusstein, Germany). The cell morphology was examined with the field-emission scanning electron microscope FE-SEM Supra 25 (Carl Zeiss, Jena, Germany) without gold coating at a low acceleration voltage of 1 kV.

### 2.4. Quantification of the Cell Area and the Cell Elongation

The cell area on the titanium arrays was quantified after 24 h. For this purpose, cultured cells were trypsinized with 0.05% trypsin/0.02% ethylenediaminetetraacetic acid (EDTA) and washed in PBS. Then the membrane of the vital cells was stained with the red fluorescent linker (PKH26 General Cell Linker Kit, Sigma Aldrich Chemie GmbH, München, Germany) for 5 min in suspension. The fluorescent dye PKH26 did not influence the cell growth of the osteoblasts - the total RNA after 7 days of cell culture remained constant (stained cells: 13.55 mg, controls: 12.37 mg). Afterwards, cells were seeded onto the titanium arrays and cultured for 24 h. After fixation with 4% paraformaldehyde (PFA), the arrays were affixed onto a slide using a double-face glue strip and the cells embedded with a cover slip. The mounting medium was prepared using 30 g glycerine (Merck, Darmstadt, Germany), 12 g polyvinylethanol (Sigma Aldrich, St. Louis, MO, USA), 0.5 g phenol (Roth, Karlsruhe, Germany) in 30 mL aqua dest. and 60 mL of 0.1 M TRIS buffer solution at pH 8.5 (Roth, Karlsruhe, Germany).

Image acquisition was performed with the confocal laser scanning microscope LSM 410 (Carl Zeiss, Jena, Germany). The cell area and the perimeter of the cell (40 cells per specimen) were measured using ImageJ version 1.43 software. The cell shape was quantifiable by calculating the form factors of each individual cell, described as 4πA/P2, where A is the cell area and P is the cell perimeter [23]. This index describes the cell shape taking its irregularity into consideration: a form factor with the value of 1 reflects a perfectly round cell whereas polygonal cells display a form factor < 1.0.

### 2.5. Flow Cytometric Measurement of Cell Proliferation

MG-63 cells on titanium arrays were cultured for 24 h and trypsinated. Cells in suspension were washed with PBS, centrifuged, and fixed with 70% ethanol overnight at −20 °C. After washing twice, cells were treated with RNase (Sigma, 1 mg/mL) at 37 °C for 20 min and incubated with propidium iodide (Sigma, 50 µg/mL). Flow cytometric measurements were performed on a flow cytometer equipped with an argon-ion laser of the wavelength 488 nm (FACSCalibur, BD Biosciences). For data acquisition, up to 20,000 events per sample (from 3 independent experiments per sample) were acquired using the CellQuest Pro 4.0.1 (BD Biosciences) software package. For data analysis of the percentage of proliferative cells, the cell cycle phases (Synthesis+Gap2/Mitosis, *i.e.*, S+G2/M) were calculated using ModFIT LT 3.0 for Power Mac G4 (BD Biosciences).

### 2.6. Microscopic Analysis of the Actin Cytoskeleton

MG-63 cells were cultured on the titanium arrays for 24 h. After fixation with 4% PFA (10 min, room temperature (RT)), cells were washed twice with PBS and permeabilized with 0.1% Triton X-100 (10 min, RT) (Merck, Darmstadt, Germany). Afterwards cells were incubated with phalloidin-TRITC (diluted 1:10) (Sigma Aldrich Chemie GmbH, München, Germany) for 30 min in the dark at RT, washed again and embedded with a cover slip in mounting medium. Actin was investigated with an inverted confocal laser scanning microscope LSM 410 (Carl Zeiss, Jena, Germany) equipped with a helium/neon-ion laser (excitation: 543 nm) and a ZEISS 63× water immersion objective (C-Apochromat 63, 1.25 W/0.17). The confocal images (512 × 512 pixel) were used for subsequent actin quantification via mathematical image processing by the FilaQuant software (University of Rostock, Institute of Mathematics, Mathematical Optimization) [24,25].

### 2.7. Actin Quantification via Mathematical Image Processing

**Figure 2:**
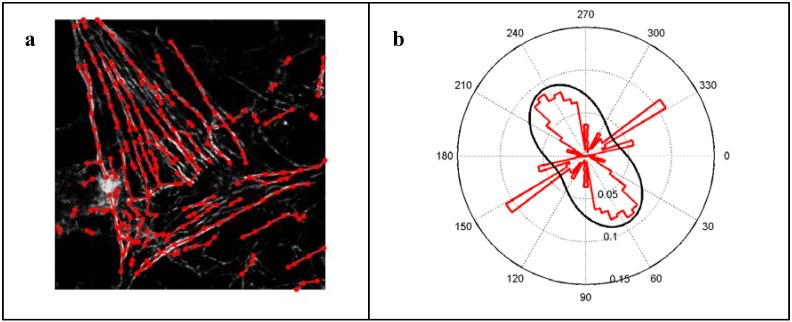
(**a**) Superposition of gray-scale image and automatically detected filaments (red). Circular markers illustrate branching points and bends. Lines connecting these markers indicate straight parts of the filament. (**b**) Polar plot of the length weighted histogram of angles of straight filament parts with the horizontal axis (red). The black line shows the fit of the von-Mises distribution on the half circle. Note that the contradiction to the assumption of one mode with the von-Mises distribution leads to a fit which is close to the uniform distribution.

In order to get an objective and efficient estimation of morphological parameters, the actin distribution was quantified by the newly developed software FilaQuant (University of Rostock, Institute of Mathematics, Mathematical Optimization) [24,25]. This software allows the automatic quantification of the cellular actin filament formation in different cell types, e.g., MG-63 cells or primary osteoblasts but also in other cells like fibroblasts or mesenchymal stem cells (MSC). It can be applied to analyze single cells as well as high density cultures. Thereby either implants with artificial surface topographies or those of clinical relevance, e.g., corundum-blasted or machined titanium surfaces including different chemical compositons are suitable for the examination by FilaQuant software. Thus actin formation could be analyzed quantitatively in a wide range of cell types in dependence on specific biomaterials’ surface characteristics, either physical modifications (e.g., the influence of biomaterials’ microtopography, like it was observed in our study) or chemical modified surfaces. The microscopic images were automatically processed in three steps: preprocessing, feature detection and quantification. Before feature detection, the main sources of errors are reduced, namely noise, by variational methods [25] and irregular background illumination by morphological white top-hat [26]. In the preprocessed image, the feature is detected by a coarse segmentation with thresholding of the digital Laplacian [26] and a fine segmentation of lines between pixels from this set. This is also called predictor-corrector principle in which an initial estimation of the unknown is used as prior knowledge for a final estimation. The corrector criterion is a sufficient mean concavity of the interpolated image relief perpendicular to the line in question. Redundancy is avoided by successive reduction of the coarse segmentation by represented pixels. The connectivity then allows for a disambiguation of superposition into representations of single filaments to measure their length. Details of this method and an evaluation of measurement errors in the use for quantification can be found in [27]. Experiments with artificial data confirm that the filament must not be too dense for this type of quantitative imaging. Especially the model of a single filament is based on a restrictive assumption. One filament is determined by a limited curvature, *i.e.*, no bend enclosing less than 135°, and the minimal in-trail curvature within all decompositions of the feature graph into edge trails. This limits accurate parameter estimation to hypotheses about the whole cell scale. Additionally the graph-based feature is evaluated for orientation preferences under circular distribution assumptions as it was proposed in Marquez *et al.* [28]. The approach is illustrated in Figure 2, including the superposition of a confocal laser-scanning microscopic image in grayscales with the automatically detected feature graph and the histogram of expected length, given angles between 0° and 180°, in red together with a fit of a von-Mises distribution. The polar plot is mirrored at the origin. All three steps are implemented in our novel software solution entitled FilaQuant, specified as a Win32 application with Embarcadero^®^ C++Builder^®^ 2010. For the quantification of the actin cytoskeleton in osteoblasts in response to a distinct surface topography, 30 cells per specimen were analyzed with FilaQuant. The resulting parameters for the description of actin filament formation were as follows: total filament length, average filament length, maximum filament length and orientation dispersion. The parameter ‘orientation dispersion’ describes the presence of a preferred orientation of the actin filaments under the von-Mises distribution assumption (Figure 2). The values range from 0°–28.65°, whereby 0° implies exactly one preferred orientation and 28.65° indicates a uniform distribution of oriented length to total length ratio.

### 2.8. Statistical Analysis

Statistical analysis was performed using SPSS-software version 15.0 for Windows (SPSS Inc., Chicago, IL, USA): Kolmogorov-Smirnov test, unpaired and paired samples *t*-test. Data were presented as a mean ± standard deviation (SD). Results of *p* < 0.05 were considered significant (denoted by: * *p* < 0.05; ** *p* < 0.01; *** *p* < 0.001).

## 3. Results

### 3.1. Morphometric Analysis of MG-63 Osteoblasts

**Figure 3:**
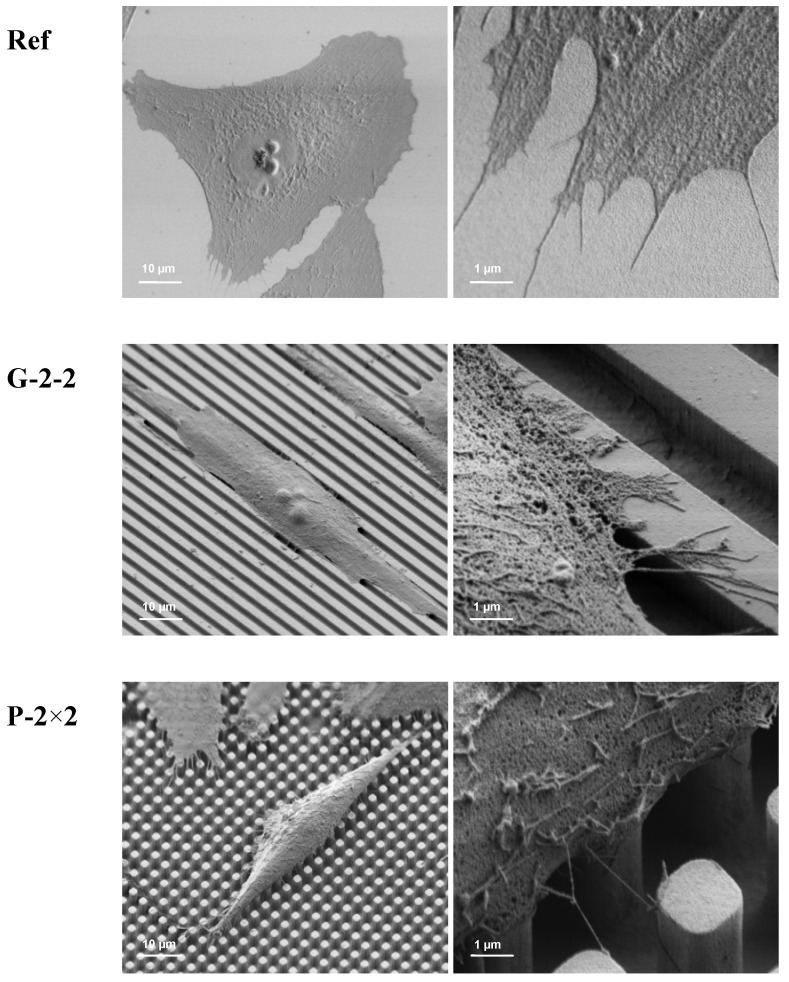

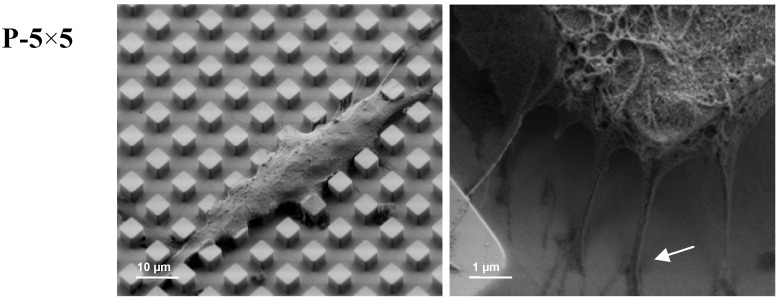
Cell morphology of MG-63 osteoblasts on planar (Ref) and geometrically microtextured titanium surfaces after 24 h. FE-SEM images reveal that on the Ref, cells exhibit a flattened phenotype and are closely attached to the surface. In contrast, on the microtextured surfaces, cell adhesion is mainly restricted to the surface plateaus. In particular, on the grooved surface G-2-2, osteoblasts do not attach to the side walls and likewise on P-2×2, and on P-5×5 only the filopods of the cells reach the bottom (arrow). Moreover, on all microtextured surfaces, cell orientation is guided by the underlying surface micropattern (contact guidance), whereas on G-2-2 osteoblasts are aligned along the grooves and on P-2×2 and P-5×5, osteoblasts predominantly orient in 45° and 90° angles to the pillars (FE-SEM Supra 25, 30°, Carl Zeiss; bars: left = 10 µm, right = 1 µm).

The morphology of MG-63 osteoblasts after 24 h cultivation time on the titanium arrays is shown in Figure 3. Cells that were grown on the microtextured samples exhibit morphological differences dependent on the underlying surface. Here MG-63 osteoblasts demonstrate an elongated phenotype and an orientation in accordance with the geometrical surface topography: In particular, the grooved surface G-2-2 induces an alignment of the osteoblasts along the ridges of the parallel grooves. The same could be observed for the pillar surfaces; here the cells orient themselves in directions of 45° and 90° angles horizontal to the pillars, also presenting topographically induced contact guidance (Figure 3). On the microtextured surfaces, the cells mainly extend on the surface plateaus. In contrast, on the planar sample (Ref), the cells show a random orientation and exhibit a flattened phenotype; they are well spread and attach to the surface with the entire cell body. Interestingly, not only the grooved microstructure is able to guide the cells but also the regular pillar structure induces a cell elongation.

**Figure 4:**
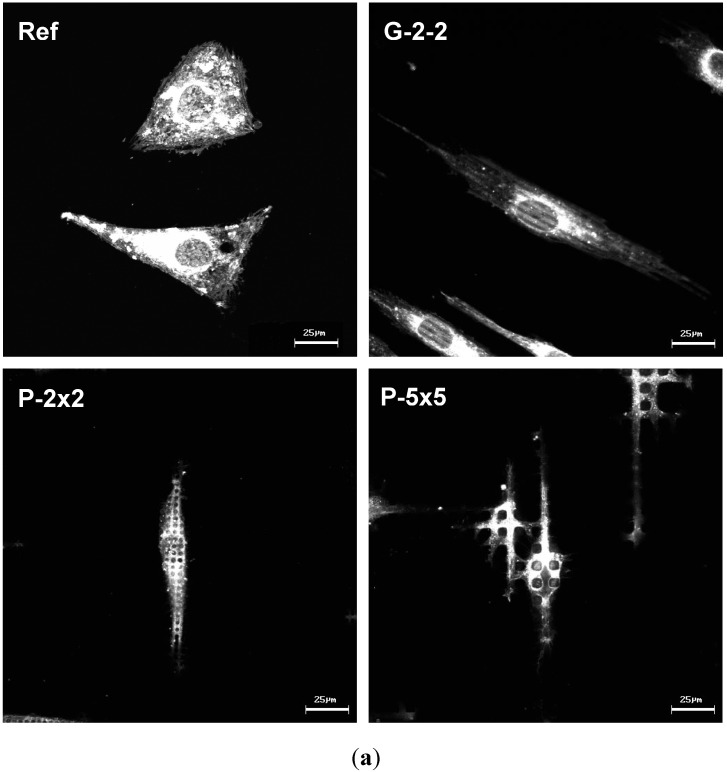

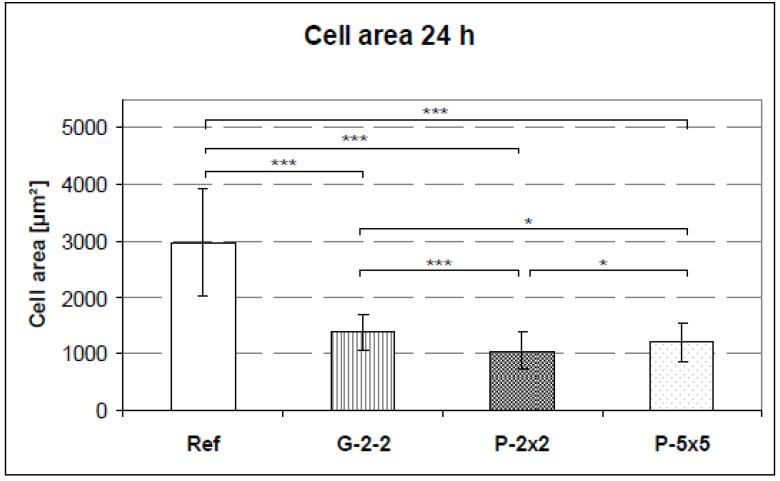
(**a**) Cell area of membrane stained MG-63 cells after 24 h. Cells grown on all microtextured surfaces exhibit a reduced and more elongated phenotype than on the planar reference (Ref). Interestingly, also pillar structures induce this elongated phenotype (LSM 410, Carl Zeiss). (**b**) Analysis of the cell area after 24 h on the microtextured surfaces compared to the planar reference (Ref). Note that cell area on all microstructures is significantly reduced, independently of grooves (G-2-2) or pillars (P-2×2, P-5×5) (mean ± SD; *p < 0.05, ***p < 0.001, unpaired *t*-test, n = 40).

Regarding the cellular spreading, the cell area of MG-63 osteoblasts on the microtextured samples is significantly decreased after 24 h and less than half compared to the reference (*p* < 0.001) (Figure 4a,b). Specifically, the cell area on the Ref amounts to 2984.2 µm², whereas on G-2-2, P-5×5 and P-2×2 it only displays values of 1383.7, 1217.4 and 1060.8 µm², respectively. Among the microstructures, the cell area is significantly lower on the pillared surfaces than on G-2-2, with the lowest value for P-2×2 (*** *p* < 0.001). We can exclude the possibility that the lower cell spreading on structured substrates is caused by cell death because no apoptotic peak appeared in the DNA histogram, as revealed by flow cytometric investigations (data not shown).

**Figure 5:**
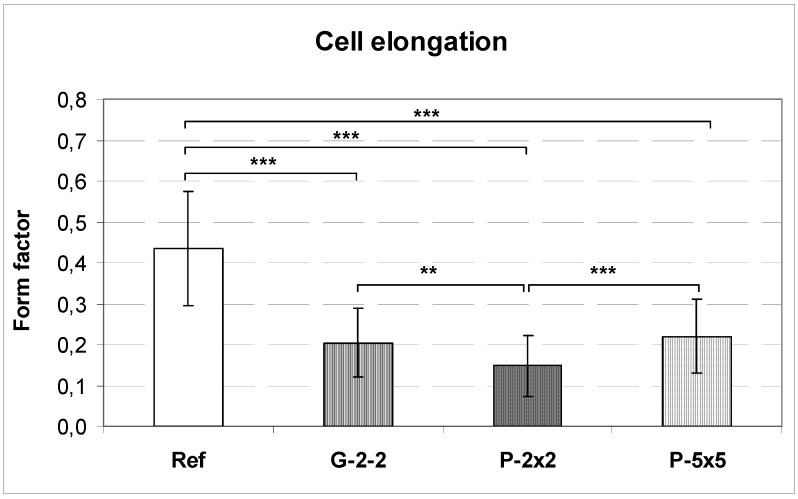
Cell elongation of MG-63 osteoblasts on microtextured titanium surfaces after 24 h. The cell shape is determined by the form factor of the cells. A form factor of 1 reflects a perfectly round cell and decreases when cells become more polygonal. Note the highest elongation for cells grown on pillars P-2×2 (mean ± SD; ** *p* < 0.01, *** *p* < 0.001, unpaired* t*-test, n = 40).

By calculating the form factors of the cells we were able to quantify the cell shape of MG-63 osteoblasts with regard to cell elongation. The form factor is high when cell shape is round (maximum value of 1) and decreases when cells become more polygonal. Based on this calculation, we revealed that MG-63 cells grown on the microstructures generally demonstrate a more elongated phenotype than on the planar surface. This is indicated by decreasing form factors of 0.44 to 0.22 to 0.2 and 0.15 for Ref, P-5×5, G-2-2 and P-2×2, respectively (Figure 5). Interestingly, the osteoblasts grown on the pillared surface P-2×2, and not those grown on the grooved surface, displayed the highest elongation and contact guidance.

### 3.2. Cell Proliferation

**Figure 6:**
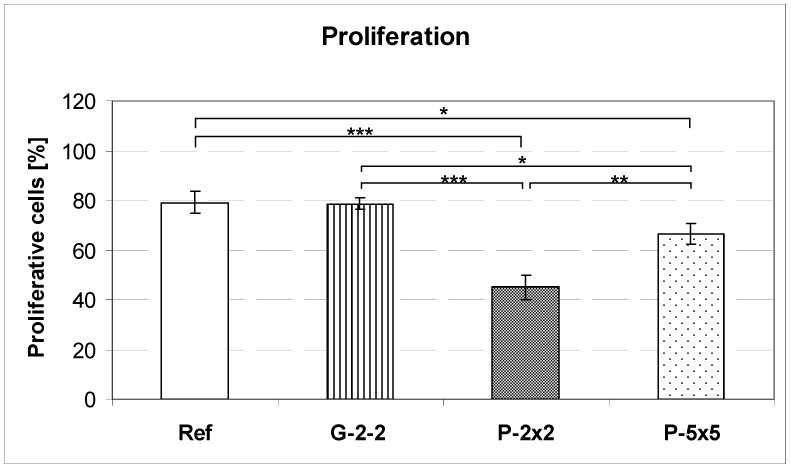
Flow cytometric analysis of the proliferative activity of MG-63 osteoblasts on microtextured titanium surfaces after 24 h. Cell proliferation on the pillared surfaces P-2×2 and P-5×5 is reduced compared to the control (Ref). In contrary, cell proliferation of MG-63 osteoblasts on the grooved surface G-2-2 is similar to that of cells grown on the planar reference (Ref) (mean ± SD; * *p* < 0.05, ** *p* < 0.01, *** *p* < 0.001, unpaired *t*-test, n = 3 à 20,000 cells) (FACSCalibur, BD Biosciences).

In order to evaluate the influence of different surface topographies on the proliferative activity of MG-63 osteoblasts, we calculated the percentage of proliferative cells after 24 h (Figure 6). On the pillared surfaces P-2×2 and P-5×5, osteoblasts display significantly lower proliferative rates of 45.1% and 66.6%, respectively in comparison to the planar control surface (79.2%), whereby the lowest number of proliferative cells is found on P-2×2. In contrast, on the microgrooved surface G-2-2 the cell proliferation (78.6%) is similar to that of cells grown on the planar control (Ref), (79.2%). In addition, on all microtextured titanium surfaces no increased apoptotic rate could be found, as revealed by the sub-G1 peak (0.3, 0.35, 0.38 and 0.28% for Ref, G-2-2, P-2×2 and P-5×5, respectively).

### 3.3. Quantification of Actin Filament Organization

**Figure 7:**
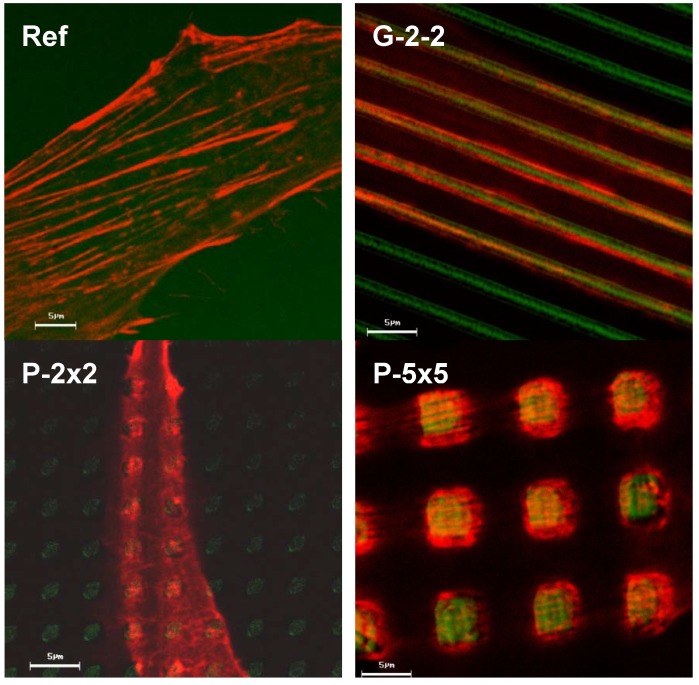
Formation of actin filaments in MG-63 osteoblasts after 24 h. On G-2-2, actin stress fibers orient themselves parallel to and along the ridges of the grooves. On the pillar structure (P-2×2, P-5×5) the actin cytoskeleton is strongly accumulated on the top of the pillars in short fibers within one cell’s dimension (LSM 410, Carl Zeiss, green: reflexion mode from the surface, red: phalloidin-TRITC for actin).

Actin is the major component of the cellular cytoskeleton and is known to play a fundamental role in diverse cellular processes which control the morphological and functional behavior of the cells. Thus, we focused on the investigation of the cellular actin cytoskeleton, for the first time including a quantification of the actin filament organization, which was carried out by a novel software solution FilaQuant. Confocal microscopy was exploited to simultaneously visualize the actin cytoskeleton and the topography of the titanium surfaces. When regarding actin distribution on the microtextured surfaces, considerable rearrangements could be revealed. On the planar reference, actin is organized typically as a network of well-defined stress fibers, spanning the entire cell body (Figure 7). On the microtextured surfaces, the actin formation adapts to the underlying surface topography. In particular, on G-2-2 there is a parallel orientation of the actin stress fibers with a periodical alignment along the ridges of the rectangular grooved structure (Figure 7). Also on the pillared surfaces P-2×2 and P-5×5 the actin distribution conforms to the pillared surface geometry. But in contrast to the planar control (Ref) as well as to the grooved array (G-2-2), on the pillared surfaces actin was not able to form stress fibers, but only short filaments, concentrating on the tops and edges of each pillar. However, the observed actin accumulation on the pillar plateaus was more obvious on P-5×5 than on P-2×2.

**Figure 8:**
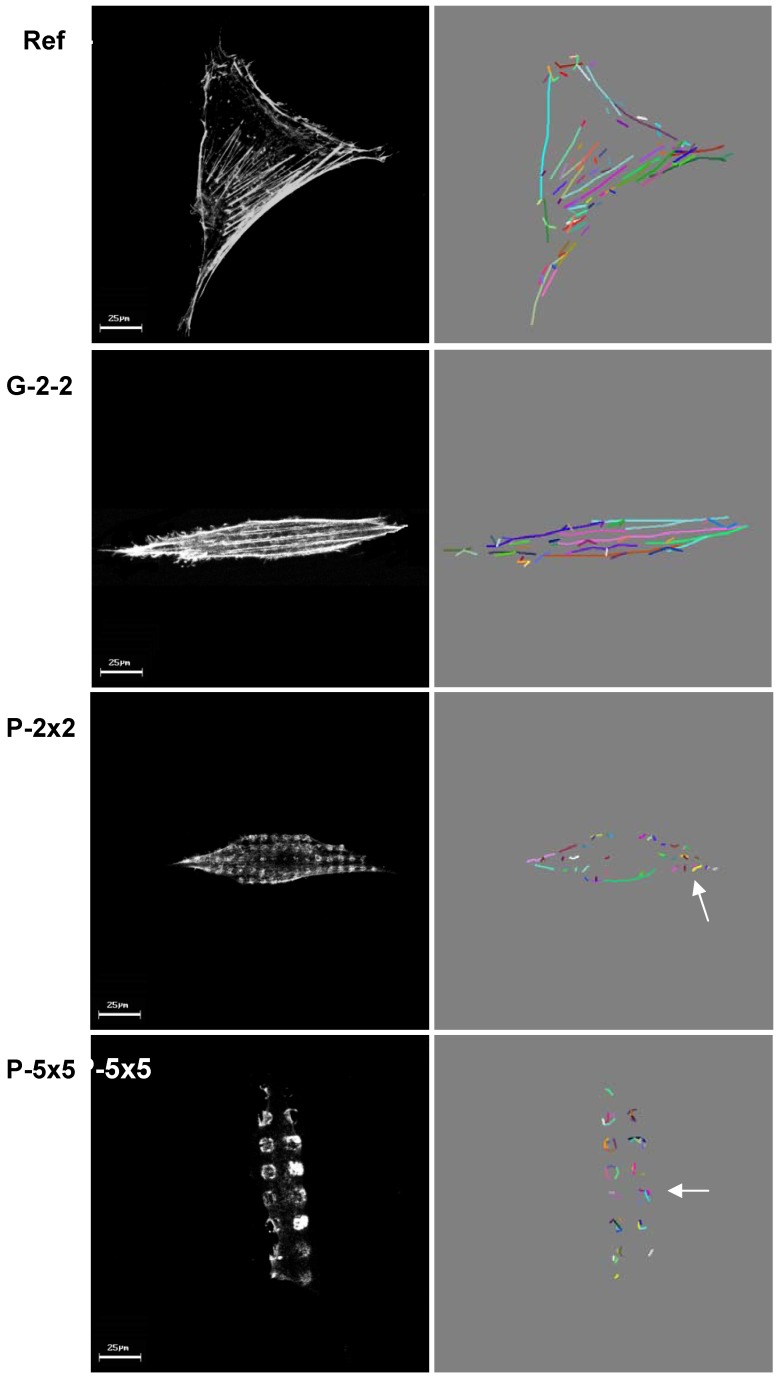
Quantification of actin filaments. left column: confocal images (LSM 410, Carl Zeiss), right column: automatically processed images obtained from FilaQuant software. Actin filaments are shown as colored lines. Note that the actin filament length is dramatically reduced (arrows) on the pillared surfaces P-2×2 and P-5×5, but cell orientation is comparable to G-2-2.

By means of the newly developed software FilaQuant for actin filament recognition, we were first able to quantify the actin organization via mathematical image processing of the confocal microscopic exposures (Figure 8). The automatic processed images represent the actin filament formation in conformance to the confocal microscopic observations and show obviously, that the average filament length on the pillared surfaces is highly reduced.

**Table 1:** Quantification of actin filament formation in MG-63 osteoblasts on microtextured titanium surfaces. Data are obtained from FilaQuant software, for automatic filament recognition, covering the majority of filaments and their branching in noisy data (images in Figure 8). Note that maximum filament length as well as average filament length on pillared surfaces P-2×2 and P-5×5 are significantly reduced compared to the Ref. Moreover the low value for orientation dispersion for cells grown on G-2-2 displays the alignment of the actin filaments along the grooves (mean ± SD, unpaired *t*-test, n = 30).

	Ref	G-2-2	P-2x2	P-5x5
**Filament number**	70.1 ± 31.4	26.0 ± 11.1 *^a^*	30.0 ± 10.6 *^a^*	33.4 ± 14.3 *^a,c^*
**Total filament length [µm]**	693.0 ± 306.9	350.8 ±205.6 *^a^*	63.5 ± 31.8 *^a,d^*	75.2 ± 36.8 *^a,d^*
**Average filament length [µm]**	9.7 ± 1.5	13.6 ± 3.8 *^a^*	2.2 ± 0.6 *^a,d^*	3.1 ± 0.4 *^a,d,e^*
**Maximum filament length [µm]**	51.5 ±11.9	54.1 ± 16.0	6.2 ± 3.5 *^a,d^*	6.7 ± 2.0 *^a,d^*
**Orientation dispersion [°]**	18.9 ±4.0	6.3 ± 2.9 *^a^*	21.5 ± 3.8 *^b,d^*	24.0 ± 3.0 *^a,d,f^*

^a^: *** *p* < 0.001 *vs.* Ref; ^b^: * *p* < 0.05 *vs.* Ref; ^c^: * *p* < 0.05 *vs.* G-2-2; ^d^: *** *p* < 0.001 *vs.* G-2-2; ^e^: *** *p* < 0.001 *vs.* P-2×2; ^f^: ** *p*<0.01 *vs.* P-2×2.

In detail, the resulted data reveals a significant decrease of the number of actin filaments on all microtextured surfaces in contrast to the planar reference (Table 1). Moreover, on all microtextured surfaces, the total filament length is significantly reduced, ranging from Ref > G-2-2 > P-5×5 > P-2×2. Thereby, maximum filament length on the planar reference is about 8-times greater than on the pillared surfaces. Also average filament length was significantly reduced on the pillared surfaces P-2×2 and P-5×5 (2.2 µm and 3.1 µm, respectively) when compared to Ref (9.7 µm).

Moreover by calculation of the orientation dispersion of the actin filaments we were able to describe the orientation of actin filaments in response to the underlying surface microtexture. On G-2-2, orientation dispersion is very low, as displayed by a value of 6.3°. This indicates that actin filaments of the cells grown on this surface tend to orient themselves in one distinct preferred direction, namely along the ridges of the grooves, as we observed from the confocal images (see Figure 7). In contrast, on the planar control (Ref), orientation dispersion increases to a value of 18.9°; despite this, the value is still much lower than for P-2×2 and P-5×5. Here the values for orientation dispersion are higher (21.5° and 24.0°, respectively). This behavior on pillared surfaces reflects the increasing loss of orientation of the shortened actin filaments and an equal distribution without any favored orientation.

## 4. Discussion

Cells are sensitive to the surface topography of a biomaterial and display a specific physiological response, involving individual, phenotypical traits dependant on that topography. However, correlative data on cell behavior in connection with the underlying physico-chemical material characteristics still remain insufficient. Only a few researchers tried to correlate the cell and the material parameters [2,29,30]. Beyond the diversity of the biological response, this could also be due to the high structural complexity of material surfaces. While most of the studies apply topographically modified surfaces, possessing random topographical features, it is a complicated undertaking to specify which structural properties of a surface are the causes of individual aspects of cell responses.

To address this, in our study we used titanium wafers with distinct, regular microtextures in order to facilitate the determination of the topographical influence on osteoblast response. By using the technology of dry etching for sample fabrication, much sharper horizontal and vertical edges of the samples were obtained in contrast to samples fabricated by photolithographic processes as they were used for previous studies [9,18].

Our morpho-functional analysis revealed that MG-63 osteoblasts exhibit individual phenotypic changes as well as functional alterations dependent on the microtopographical features of the titanium surfaces. We revealed that cells arranged their morphology and modulate their spreading in accordance with the underlying surface structure.

In particular, osteoblasts that were grown on the planar titanium as a control surface displayed a polygonal cell shape, typical for osteoblasts [17]. In contrast, on the microtextured surfaces cells showed topography-induced contact guidance, while exhibiting an elongated phenotype, which was expressed by lower form factors. These observations support other studies which reported that a geometrically oriented microtopography of titanium surfaces orients MG-63 cells and thus alters their morphology, even resulting in a very elongated phenotype [6,18]. In particular, Sjöström *et al.* [31] reported that nanopillared titanium arrays induce an elongation of MG-63 cells whereas increasing pillar height leads to higher cell elongation and moreover to reduced spreading of the MG-63 cells. Also, in our study cell spreading of MG-63 osteoblasts was highly reduced on the microstructures after 24 h when compared to the planar surface. Interestingly, on all microtextured surfaces, regardless of their distinct surface designs (grooved *vs.* pillared), cells displayed less than half of the cell area when compared with the planar reference. Apart from this, among the microtextured arrays G-2-2, P-2×2 and P-5×5 only slight differences in the cell area exist. This showed that cellular spreading was not affected as much by the different surface designs demonstrated here (kind and microdimension) but rather by the surface classification smooth *vs.* microtextured, indicating that this might possess the superordinate surface characteristic.

To explain the decrease of cell spreading on the microtextured surfaces in comparison to the planar surface might involve more than one cause. On the one hand, it could be due to the decreased cell attachment area which was mainly restricted to the plateaus of the pillars and grooves and led to a reduced adhesion capacity e.g., on pillars [9]. On the other hand, topographically modified surfaces probably hold distinct physico-chemical properties due to the surface geometry itself e.g., differences in surface wettability or surface energy, which are known to reduce cell spreading as we have shown in our previous study [6,9]. Moreover, these distinct surface characteristics of microtextured surfaces could have an unfavorable impact on the quality and/or quantity of protein adsorption to the surface which also could be responsible for lower cell spreading.

Concerning cell growth on the pillared surfaces we found diminished cell proliferation after 24 h in comparison to the planar control. In contrast, the cell proliferation of osteoblasts grown on the grooved titanium array was nearly the same as that of cells grown on the planar reference. Note that no apoptosis-inducing effect was observed. Hunter *et al.* reported that one major regulator of cell proliferation is the cell shape; cells which only spread a little display lower proliferation rates than those with greater cell areas [32]. This might be an explanation for our observations on the pillared surfaces P-2×2 and P-5×5 because here the decrease of cell spreading was accompanied by a diminished proliferative activity. In contrast, on the grooved surface G-2-2 we did not find such a relationship between cell area and cell proliferation: although the cell area is reduced, the S+G2/M phase-values are comparable to the planar surface. In consequence, the observation of Hunter *et al.* seems not to be sufficient to explain the decrease of the proliferation of MG-63 osteoblasts grown on the pillared surfaces. Hence, this means that not only differences in cell morphology (spreading) are solely responsible for alterations in cell function, but that there are additional causes which have to be taken into consideration.

Among others, one possible factor determinant for cell physiology might be the actin cytoskeleton, since it is known that its arrangement and distribution is crucial for numerous fundamental cellular functions [9,11,17,33]. In our investigation, confocal microscopy showed that on the grooved microstructure, actin formed well defined stress fibers like it also was observed for the cells that were grown on the planar control. In contrast, on the pillared surfaces a strong rearrangement of the actin cytoskeleton was revealed. Here actin was not organized in typical stress fibers but only in short fragments clustering on the top and edges of the pillars. This observation was consistent with our previous study dealing with titanium-coated SU-8 micropillars (obtained from photolithographic etching with the photoresist SU-8); here we also demonstrated an accumulation of shortened actin filaments on the pillar plateaus and edges [9]. Moreover current investigations on primary bone cells show similar phenotypic actin rearrangements on the micropillars (unpublished results).

Additionally, in this study for the first time we were able to quantify the observed differences in actin filament formation by means of our novel software, specially developed for automatic filament recognition. Using this software we were able to collect data-based evidence for the observed structural actin rearrangements. Thus the development of this new software represents a significant effort for biological data quantification, which enabled the examination of actin filament formation in different cell types, not only osteoblasts, on a diverse range of implant surfaces, e.g., the average actin filament length of MG-63 cells on Ti machined [17] is nearly 4-times higher (8.2 µm) *vs.* Ti corundum-blasted (2.3 µm) after 24 h. Thus, the application of FilaQuant is independent of the cell type as well as the material surface. The only precondition for an exact analysis is a visible cytoskeleton.

Moreover to our knowledge it is the first published software for automatic actin quantification. Indeed, the obtained data revealed a significant decrease in the total number of actin filaments as well as a significantly reduced average filament length on the pillared surfaces, correlating with the lower proliferation rates on these surfaces. In contrast, on the grooved surface the average actin filament length was similar to that of cells grown on the planar control, where the proliferative rate was shown to be nearly the same. In consequence, we assumed the rearrangement of the actin cytoskeleton to be the cause of the reduced proliferation of osteoblasts on the pillared surface and not the overall cell morphology.

Moreover, by confocal observation of the actin cytoskeleton we revealed an adaptation of actin filament organization correlating to the surface microtexture, as displayed by the parallel alignment of actin filaments strictly along the ridges of the grooves on G-2-2 as well as the actin accumulation towards the top of each micropillar for P-2×2 and P-5×5; this was also found on SU-8 pillars 5 × 5 in our earlier study [9]. Consequently we clearly demonstrated that not only the morphology of MG-63 osteoblasts is altered dependent on surface topography, but also actin architecture, which mimics the underlying surface structure of pillars and grooves. Curtis *et al.* [34] also observed a condensation of the actin cytoskeleton of human macrophages and platelets in correspondence to the nanopatterning of polymer substrates (nanogrooves and nanopits). He proposed that the topographical-induced imprinting of the cell’s plasmalemma would thereby provide an ordered array of anchor points for the actin cytoskeleton and thus might be responsible for the effects of ordered nanotopography on cytoskeletal organization [34].

Besides, we conjecture local physico-chemical properties at the edges and ridges of the surface which are responsible for this actin ‘mimicry’ rather than an altered protein adsorption, because we observed the same phenomenon in serum-free culture conditions (unpublished results). This might also be supported by a study of Walboomers *et al.* [35] who investigated the attachment of fibroblasts to smooth and microgrooved (1–20 µm wide, 0.5–5.4 µm deep) polystyrene substrates. He reported that surface discontinuities, such as edges or ridges, display distinct mechanical forces which cause the cells to reshape their actin filaments there and adjust to the substrate topography. Moreover, contact guidance is caused by these mechanical forces on the cells’ filopodia [35].

Although this could be the reason for the observed contact guidance on the microgrooved surface, as is also demonstrated in our study, it does not seem to be totally valid to explain the cell orientation detected by citing the micropillared surfaces because, interestingly, on these surfaces the elongation of osteoblasts occurred without any accompanying actin alignment.

This leads us to another interesting point we should elucidate: the relationship between cell orientation and actin organization.

Many studies propose that actin polymerization traits are causative in the length control of cells as well as in contact guidance [14,36,37]. The authors conclude that cell orientation is determined by the formation of the actin cytoskeleton, whereas the elongation conforms to the orientation of actin fibers along the polarized cell axis. In our study we can refute this theory, verified by the observed cell elongation but inhibited actin formation of osteoblasts grown on the pillared surfaces. Thus, it seems that the occurrence of cell orientation is independent of a respective alignment of actin filaments, which is contradictory to the postulation by others. Hence, our results demonstrate that we probably have to reconsider and expand our view of the mechanisms responsible for the orientation of cells.

Taken together, from our morphometric analysis of osteoblasts on microgrooved and micropillared surfaces, we obtained new insights into cell-interface interactions, especially with regards to correlations of distinct cellular parameters, e.g., form factor, actin filament length or total actin number. In particular, we elucidated relationships between cell shape and cell orientation as well as the impact of intracellular structures, namely the arrangement of actin cytoskeleton on cell physiological processes, e.g., the cell cycle phases.

The novel software FilaQuant introduced here to quantify the actin cytoskeleton of one cell in response to the underlying surface microstructure is an important tool to obtain quantitative results from confocal microscopic images and enhance the current state of data for the correlation of cellular parameters with distinct material surface characteristics.

## 5. Conclusions

In this investigation on MG-63 osteoblasts’ behavior on regular microtextured titanium arrays, we focused on the morphometric detection and quantification of the biomaterial influence on the cell phenotype and the actin cytoskeleton. We were able to find out that (i) the elongation of cells is not driven by the actin cytoskeleton on pillared structures, (ii) the actin cytoskeleton seems to be responsible for the cell proliferation, and (iii) pillars induce equal contact guidance to grooves. By means of our novel software FilaQuant, especially developed for mathematical analysis of the actin cytoskeleton from microscopic images, we were first able to quantify the observed alterations in actin filament organization and correlate the data: average filament length, total filament length, filament number and orientation dispersion with material characteristics regarding surface microdimensions. With this study we demonstrated how new tools for biomedical image processing could open new possibilities for data correlation in investigations on the cell-biomaterial dialogue. These challenging approaches will contribute to an elucidation of the impact of physico-chemical surface modifications on cellular physiology and so benefit the improvement of implant design in the future.

## References

[1] Jäger M., Zilkens C., Zanger K., Krauspe R. (2007). Significance of nano- and microtopography for cell-surface interactions in orthopaedic implants. J. Biomed. Biotechnol..

[2] Biggs M.J.P., Richards R.G., Gadegaard N., Wilkinson C.D.W., Dalby M.J. (2007). The effects of nanoscale pits on primary human osteoblast adhesion formation and cellular spreading. J. Mater. Sci. Mater. Med..

[3] Nebe J.B.G., Luethen F., Lange R., Beck U. (2007). Interface interactions of osteoblasts with structured titanium and the correlation between physico-chemical characteristics and cell biological parameters. Macromol. Biosci..

[4] Passeri G., Cacchioni A., Ravaneti F., Gall C., Elezi E., Macaluso G.M. (2010). Adhesion pattern and growth of primary human osteoblastic cells on five commercially available titanium surfaces. Clin. Oral Implants Res..

[5] Zinger O., Zhao G., Schwartz Z., Simpson J., Wieland M., Landolt D., Boyan B. (2005). Differential regulation of osteoblasts by substrate microstructural features. Biomaterials.

[6] Ismail F.S.M., Rohanizadeh R., Atwa S., Mason R.S., Ruys A.J., Martin P.J., Bendavid A. (2007). The influence of surface chemistry and topography on the contact guidance of MG63 osteoblast cells. J. Mater. Sci. Mater. Med..

[7] Curtis A.S., Wilkinson C.D. (1998). Reactions of cells to topography. J. Biomater. Sci. Polym..

[8] Zhao G., Zinger O., Schwartz Z., Wieland M., Landolt D., Boyan B.D. (2006). Osteoblast-like cells are sensitive to submicron-scale surface structure. Clin. Oral Implants Res..

[9] Matschegewski C., Staehlke S., Löffler R., Lange R., Chai F., Kern D., Beck U., Nebe J.B. (2010). Cell architecture-cell function dependencies on titanium arrays with regular geometry. Biomaterials.

[10] Yang J.-Y., Ting Y.-C., Lai J.-Y., Liu H.-L., Fang H.-W., Tsai W.-B. (2009). Quantitative analysis of osteoblast-like cells (MG63) morphology on nanogrooved substrata with various groove and ridge dimensions. J. Biomed. Mater. Res. A.

[11] Stricker J., Falzone T., Gardel M.L. (2010). Mechanics of the F-actin cytoskeleton. J. Biomech..

[12] Clark E.A., Brugge J.S. (1995). Integrins and signal transduction pathways: The road taken. Science.

[13] Mooney D.J., Langer R., Ingber D.E. (1995). Cytoskeletal filament assembly and the control of cell spreading and function by extracellular matrix. J. Cell Sci..

[14] Kharitonova M.A., Vasiliev J.M. (2003). Length control is determined by the pattern of cytoskeleton. J. Cell Sci..

[15] Uggeri J., Guizzardi S., Scandroglio R., Gatti R. (2010). Adhesion of human osteoblasts to titanium: A morpho-functional analysis with confocal microscopy. Micron.

[16] Lange R., Lüthen F., Beck U., Rychly J., Baumann A., Nebe J.G.B. (2002). Cell-extracellular matrix interaction and physico-chemical characteristics of titanium surfaces depend on the roughness of the material. Biomol. Eng..

[17] Lüthen F., Lange R., Becker P., Rychly J., Beck U., Nebe J.G.B. (2005). The influence of surface roughness of titanium on ß1- and ß3-integrin adhesion and the organization of fibronectin in human osteoblastic cells. Biomaterials.

[18] Lange R., Elter P., Biala K., Matschegewski C., Stählke S., Löffler R., Fleischer M., Nebe J.B., Kern D., Beck U. (2010). Titanium surfaces structured with regular geometry—Material investigations and cell morphology. Surf. Interface Anal..

[19] Lu X., Leng Y. (2003). Quantitative analysis of osteoblast behavior on microgrooved hydroxyapatite and titanium substrata. J. Biomed. Mater. Res. A.

[20] Beil M., Braxmeier H., Fleischer F., Schmidt V., Walther P. (2005). Quantitative analysis of keratin filament networks in scanning electron microscopy images of cancer cells. J. Microsci..

[21] Eberly D., Gardner R.B., Morse B.S., Pizer S.M., Scharlach C. (1994). Ridges for image analysis. J. Math. Imag. Vis..

[22] Lärmer F., Schilp A. (1994). Method of Anisotropically Etching Silicon. German Patent No..

[23] Senju Y., Myata H. (2009). The role of actomyosin contractility in the formation and dynamics of actin bundles during fibroblast spreading. J. Biochem..

[24] Birkholz H., Matschegewski C., Nebe J.B., Engel K. (2010). Quantification of Actin Filament Organization by Estimating Graph Structures in Confocal Microscopic Images. Proceedings of the World Congress on Medical Physics and Biomedical Engineering.

[25] Birkholz H. (2011). A unifying approach to isotropic and anisotropic total variation denoising models. J. Comput. Appl. Math..

[26] Gonzalez R.C., Woods R.E. (2008). Digital Image Processing.

[27] Birkholz H. Extracting the Ridge Set as a Graph for Quantification of Actin Filaments Obtained by Focal Laser Scanning Microscopy. Proceedings of the SPIE 8000.

[28] Marquez J.P. (2006). Fourier analysis and automated measurement of cell and fiber angular orientation distributions. Int. J. Solids Struct..

[29] Anselme K., Bigerelle M. (2006). Modeling approach in cell/material interactions studies. Biomaterials.

[30] Giljean S., Ponche A., Bigerelle M., Anselme K. (2010). Statistical approach of chemistry and topography effect on human osteoblast adhesion. J. Biomed. Mater. Res. A.

[31] Sjöström T., Lalev G., Mansell J.P., Su B. (2011). Initial attachment and spreading of MG63 cells on nanopatterned titanium surfaces via through-mask anodization. Appl. Surf. Sci..

[32] Hunter A., Archer C.W., Walker P.S., Blunn G.W. (1995). Attachment and proliferation of osteoblasts and fibroblasts on biomaterials for orthopaedic use. Biomaterials.

[33] Hayes J.S., Khan I.M., Archer C.W., Richards R.G. (2010). The role of surface microtopography in the modulation of osteoblast differentiation. Eur. Cell Mater..

[34] Curtis A.S., Dalby M.J., Gadegaard N. (2006). Nanoprinting onto cells. J. R. Soc. Interface.

[35] Walboomers X.F., Monaghan W., Curtis A.S., Jansen J.A. (1999). Attachment of fibroblasts on smooth and microgrooved polystyrene. J. Biomed. Mater. Res..

[36] Anselme K., Bigerelle M., Noël B., Iost A., Hardouin P. (2002). Effect of grooved titanium substratum on human osteoblastic cell growth. J. Biomed. Mater. Res..

[37] Krzysiek-Maczka G., Michalik M., Madeja Z., Korohoda W. (2010). Involvement of cytoskeleton in orientation of cell division in contact guided cells. Folia Biol. (Krakow).

